# Long-Term Effects of (–)-Epigallocatechin Gallate (EGCG) on Pristane-Induced Arthritis (PIA) in Female *Dark Agouti* Rats

**DOI:** 10.1371/journal.pone.0152518

**Published:** 2016-03-29

**Authors:** Anna Leichsenring, Ingo Bäcker, Paul G. Furtmüller, Christian Obinger, Franziska Lange, Jörg Flemmig

**Affiliations:** 1 Fraunhofer Institute for Cell Therapy and Immunology (IZI) Leipzig, Perlickstraße 1, 04103 Leipzig, Germany; 2 Department of Chemistry, Division of Biochemistry, University of Natural Resources and Life Sciences (BOKU) Vienna, Muthgasse 18, 1190 Vienna, Austria; 3 Institute for Medical Physics and Biophysics, Medical Faculty, University of Leipzig, Härtelstraße 16–18, 04107 Leipzig, Germany; 4 Translational Centre for Regenerative Medicine (TRM) Leipzig, Philipp-Rosenthal-Str. 55, 04103 Leipzig, Germany; University of Leuven, Rega Institute, BELGIUM

## Abstract

Rheumatoid arthritis (RA)—a widespread chronic inflammatory disease in industrialized countries—is characterized by a persistent and progressive joint destruction. The chronic pro-inflammatory state results from a mutual activation of the innate and the adaptive immune system, while the exact pathogenesis mechanism is still under discussion. New data suggest a role of the innate immune system and especially polymorphonuclear granulocytes (PMNs, neutrophils) not only during onset and the destructive phase of RA but also at the chronification of the disease. Thereby the enzymatic activity of myeloperoxidase (MPO), a peroxidase strongly abundant in neutrophils, may be important: While its peroxidase activity is known to contribute to cartilage destruction at later stages of RA the almost MPO-specific oxidant hypochlorous acid (HOCl) is also discussed for certain anti-inflammatory effects. In this study we used pristane-induced arthritis (PIA) in *Dark Agouti* rats as a model for the chronic course of RA in man. We were able to shown that a specific detection of the HOCl-producing MPO activity provides a sensitive new marker to evaluate the actual systemic inflammatory status which is only partially detectable by the evaluation of clinical symptoms (joint swelling and redness measurements). Moreover, we evaluated the long-term pharmacological effect of the well-known anti-inflammatory flavonoid epigallocatechin gallate (EGCG). Thereby only upon early and continuous oral application of this polyphenol the arthritic symptoms were considerably diminished both in the acute and in the chronic phase of the disease. The obtained results were comparable to the treatment control (application of methotrexate, MTX). As revealed by stopped-flow kinetic measurements, EGCG may regenerate the HOCl-production of MPO which is known to be impaired at chronic inflammatory diseases like RA. It can be speculated that this MPO activity-promoting effect of EGCG may contribute to the pharmacological mode of action of this polyphenol.

**Highlights**

- Epigallocatechin gallate (EGCG), upon early and continuous oral application, considerably attenuates the symptoms in *Dark Agouti* rats with pristane-induced arthritis (PIA)
- Arthritic symptoms are not only dampened in the acute but also in the chronic phase of the disease, which means a lower risk for the development of chronic recurring joint inflammation
- The therapeutic effect is comparable to the early injection of methotrexate (MTX) and is not observed upon late oral application or injection of EGCG
- Stopped-flow kinetic measurements show that epigallocatechin (EGC) derived from EGCG exhibits a considerable activity with Compounds I and II of myeloperoxidase (MPO)
- It can be guessed that the reactivation of the chlorinating MPO activity by EGCG may contribute to the anti-inflammatory effect of the polyphenol

## Introduction

### 1.1 Rheumatoid arthritis in man

Rheumatoid arthritis (RA) is a common chronic inflammatory autoimmune disease in industrialized countries and is characterized by a persistent inflammation of joints and leads to the progressive destruction of cartilage and bone [1–3]. The exact etiology of RA is still unclear [3–5] although several endogenous (e.g. genetics, age, sex) and exogenous (e.g. smoking habits, social status) factors are known [2,4]. During the onset of RA an acute inflammation of the synovial lining (synovitis) leads to an extensive expansion of the corresponding cells (pannus formation) and the massive infiltration of leukocytes of the innate immune system (neutrophils and monocytes) into the synovial fluid [4,6]. This clinical phase is accompanied by local (pain, swelling and redness of the joints) and systemic (e.g. elevated acute phase protein levels in blood) inflammatory symptoms [2,3,6]. The chronification of RA is supposed to be mainly driven by T lymphocytes and other components of the adaptive immune system [2,3]. In fact, as shown in animal models for RA, by transferring activated CD4^+^ T cells from individuals with arthritis the disease was induced in healthy animals without further priming [3,7]. It is hypothesized that these auto-reactive immune cells create a pro-inflammatory cytokine milieu (elevated IL-6 and TNFα levels) which leads to the recruitment of further neutrophils, monocytes and macrophages [2,3,8]. The latter are regarded as effector cells responsible for cartilage and bone destruction in RA via degradative enzymes (e.g. proteases) and reactive oxygen species (ROS) [2,4].Thus the pathogenesis of RA is characterized by a chronic inflammation resulting from a mutual interaction between leukocytes of the innate and adaptive immune system [3,4,9]. Thereby the former seem to play an important role during all stages of the disease [10] as the direct therapeutic targeting of T cells often shows only limited effects in RA [4].

### 1.2 Neutrophils in rheumatoid arthritis

Neutrophils (polymorphonuclear leukocytes, PMNs), the most abundant immune cells in the blood and the first leukocytes recruited during inflammation, are essentially involved in the pathology of RA [6,9,11]. Already during onset of the disease huge amounts of these cells (up to 10^8^/ml) can be found in the synovial fluid joint space at the pannus cartilage border [6,11]. There they contribute to the creation of pro-inflammatory conditions, the activation of further leukocytes from the innate and acquired immune system as well as to cartilage and bone destruction at later stages of RA [6,9]. The latter is mainly mediated by proteases and ROS-producing enzymes like NADPH oxidase (O_2_^•-^ generation) and myeloperoxidase (MPO, HOCl production) [6,9,10] which are released by pro-inflammatory stimulated neutrophils [11].PMNs also play manifold roles in the chronification of RA [12–14]: The solidification of the disease includes autoimmune aspects as evidenced by the fact that B cell-derived rheumatoid factors (antibodies against the Fc part of IgG antibodies) and antibodies against citrullinated proteins are key markers of its pathogenesis [1,2,15]. The resulting circulating immune complexes trigger PMNs to release pro-inflammatory mediators that contribute to the chronification of the joint inflammation [10,16]. Moreover the formation of nuclear extracellular traps (NETs) by stimulated neutrophils may provide a further source for neoepitopes (e.g. citrullinated proteins) which contribute to the loss of immune tolerance during the chronification of RA [9,17]. Thus neutrophils are key players during the onset, chronification and effector phase of RA [11,18,19]. In fact, classical anti-rheumatic drugs like methotrexate (MTX) often lead to a significant reduction of the neutrophil load in arthritic joints by suppressing neutrophil chemotaxis [16,20,21] while B- and T-cells are an unlikely target of MTX treatment [21]. Yet a possible role of the almost neutrophil-specific enzyme myeloperoxidase (MPO) in the disease is still not clear [17,20]. In order to investigate the role of MPO in RA a suitable small laboratory animal model for this disease is necessary which reflects the chronic course of the disease in humans.

### 1.3 Animal models for rheumatoid arthritis

RA was classically induced in small laboratory animals (especially mice and rats) by applying complete Freund's adjuvant (CFA), assuming that the immunogenic part (inactivated *Myobacteria*) leads to the onset of the disease while the oil-based adjuvant is responsible for the boost of the disease [7]. The same holds for collagen-induced arthritis (CIA) in mice, where CFA and collagen type II is injected [3,15]. Yet, as shown e.g. by pristane-induced arthritis (PIA) in rats, the sole application of a non-immunogenic adjuvant is sufficient to induce arthritic symptoms in the animals [8,22,23]. Moreover, the latter animal model turned out to reflect not only the symptoms (e.g. pannus formation) and the immunological processes (e.g. PMN infiltration) of RA in man but also the chronic course of the disease as a recurring joint swelling is induced by a single injection of the mineral oil [7,8,22]. Moreover, unlike for e.g. CIA, no additional stimulus (boost) is needed in PIA [3].

### 1.4 Treatment of rheumatoid arthritis

RA is classically treated with steroidal (e.g glucocorticoids) and non-steroidal anti-inflammatory (NSAIDs, e.g. sulfasalazine) drugs to dampen the underlying chronic inflammatory processes [2,24,25]. Yet these drugs only decrease the inflammatory response but have no influence on the chronic course of the disease [24]. Therefore, therapeutic strategies usually combine the treatment with the above mentioned drugs with the application of disease-modifying anti-rheumatic drugs (DMARDs). Among these non-biologicals (e.g. MTX) and biologicals (e.g. TNFα inhibitors or IL-1 receptor antagonists) are distinguished [2,16,26]. Yet while these DMARDs were shown to reduce the inflammatory response both in human patients and in rodent models of RA, the underlying mechanisms of these drugs are sometimes not completely understood [2,6,21]. Moreover they often exhibit weak long-term therapeutic effects and do not always lead to a remission of the arthritic symptoms [16,21,27]. This led to a growing interest in alternative RA therapeutic options including herbal therapies [26].

As shown for several animal RA models, the natural compound epigallocatechin gallate (EGCG) also attenuates arthritic symptoms by suppressing the underlying chronic inflammation [26]. Yet again while EGCG is generally known for its anti-oxidative and anti-inflammatory effects, the exact mode of action of this flavonoid in RA is still under discussion [26]. Most probably this most abundant green tea polyphenol (GTP) mainly acts by suppressing early inflammatory events, suggesting an effect on the cells of the innate immune system [28,29]. In fact, the flavonoid has been shown to suppress pro-inflammatory IL-6 signalling, both in cellular *in vitro*-models as well as in adjuvant-induced arthritis in rats [30]. Clinical studies on RA patients have shown that a decrease of IL-6 serum levels under DMARD treatment correlates with a long-term reduction of joint inflammation [31]. A combined application of MTX and EGCG also showed beneficial effects in a comparable animal model of RA and suggests EGCG as a potent immune-modulatory agent [32].

## Material and Methods

### 2.1 Materials

Female *Dark Agouti* (DA) rats used in this study were obtained from Janvier Labs, St. Berthevin Cedex, France at an age of eight weeks. Pristane (purity: ≥ 98%) for arthritis induction was obtained from Sigma-Aldrich, Taufkirchen, Germany. Isoflurane (purity: 100% v/v) for anaesthetisation was purchased from Abbott, Wiesbaden, Germany. Epigallocatechin gallate (EGCG) (purity: 99.52%) was obtained from Chengdu Biopurify Phytochemicals Ltd., Chengdu, China and Methotrexate (TEVA GmbH, Ulm, Germany) was prepared by the pharmacy of hospital Klinikum St. Georg Leipzig as a ready-to-use solution of 0.05 mg/ml. Isotonic NaCl solution for the preparation of EGCG injection solutions was obtained from Fresenius Medical Care, Bad Homburg, Germany. Heparin used as an anti-coagulant during blood sampling was purchased from Rotexmedica, Trittau, Germany. Phosphate buffered saline (PBS) and Hanks balanced salt solution (HBSS) used during blood purification were obtained from amresco LLC, Solon, OH, USA and from Sigma-Aldrich, Taufkirchen, Germany, respectively. APF for MPO activity staining was purchased from Cayman Chemical Company, Ann Arbor, MI, USA. Alpha 1-acid glycoprotein (α1AGP) was quantified by using an ELISA-Kit from Life Diagnostics, West Chester, PA, USA. Materials for the preparation and staining of tissue slices were obtained from Merck Millipore, Darmstadt, Germany (Osteosoft®, paraffin, nuclear fast red, tungstophosphoric acid, Orange G, ethanol), Carl Roth, Karlsruhe, Germany (formaldehyde, Euparal), Waldeck GmbH, Münster, Germany (Aniline blue) and AppliChem, Darmstadt, Germany (isopropyl alcohol), respectively.Highly purified human myeloperoxidase for the stopped-flow kinetic measurements was obtained from Planta Natural Products (http://www.planta.at). Its concentration was determined using ε_430_ = 91,000 M^-1^ s^-1^ per haem [33]. Hydrogen peroxide as a 30% solution was obtained from Sigma-Aldrich, Deisenhofen, Germany. Stock solutions of H_2_O_2_ were prepared in Millipore water, stored at 4°C and determined spectroscopically immediately prior to use by using ε_230_ = 74 M^-1^ s^-1^ [34]. Epigallocatechin from green tea (EGC, E3768, purity ≥ 95%) was also purchased from Sigma-Aldrich. Stock solutions (15.5 mM) of the compound were prepared in 50% dimethyl sulfoxide (DMSO). The final DMSO concentration during the stopped-flow kinetic measurements did not exceed 0.01%.

### 2.2 Animals

The rats were earmarked for identification and housed in a climate-controlled room on a 12–12 hours light-dark cycle. Food and water were available ad libitum. Health status of the rats was monitored daily and weight was controlled at least once a week. The animal study took place at a certified laboratory animal facility (Medical-Experimental Centre, University of Leipzig, Leipzig, Germany) and all procedures were approved by the local animal usage committees according to German guidelines on animal care and use (Landesdirektion Sachsen, Referat 24, registration number: TVV13/13). At the end of the study on d155 the animals were sacrificed by carbon dioxide inhalation.

### 2.3 Arthritis induction, pharmacological treatment and arthritis scoring

After two weeks of acclimatisation, 150 μl pristane were injected intra-dermally into the tail base of the rats [7,8,22] under anaesthesia induced by an oxygen/nitrous oxide mixture (2:1 v/v) containing 3–4% isoflurane. An onset of the disease was assumed when the animals reached an arthritis score of one. The pharmacological treatment started either on day 1 after pristane injection (early treatment), or after the individual animal reached a score of five (late treatment). The animals were either treated by intra-peritoneal (i.p.) injection of 0.05 mg/kg methotrexate (MTX) [35], i.p. injection of EGCG (10 mg/kg) [23], or oral application (p.o.) of the latter compound via the drinking water (0.1% w/v) [28,29]. Thereby for the i.p. administration of EGCG the compound was dissolved in physiological NaCl solution. Both for EGCG and MTX the injection was body weight adjusted: 1 μl was injected per g body weight on five subsequent days. In the saline-treated positive control physiological NaCl solution was injected (5x, starting on d0). For the oral treatment with EGCG the compound was freshly dissolved each day in tap water and applied throughout the study. Per cage (five rats) 200 ml drinking solution were prepared. During the experiment the swelling and redness of the paws as an arthritic symptom was scored thrice a week in the acute phase (d7–d42) and twice a week during the chronic phase (d43–d155) [16]. The weight of the animals was determined one (chronic phase) to three (acute phase) times a week [16]. The arthritic score was determined according to a well-established advanced scoring system [5]. Briefly, all paws were inspected separately whereby every swollen and red toe/knuckle yielded a score of 1 and a swollen and red mid-paw or ankle had a score of 5. Thus, for each paw a maximum score of 15 was possible, yielding to a maximum score of 60 for one animal. The scoring was performed under blinded conditions. From the obtained animal-specific values the mean and the standard error of mean was calculated for each experimental group. Mean and standard error of mean values were also calculated for each experimental group from the individual weight of each animal at a given time point. Due to the fact that the obtained scoring data showed no Gaussian distribution, for comparisons between the experimental groups the Kruskal-Wallis test was used for analysis, a non-parametric method for comparison between more than two groups [36]. Thereby p ≤ 0.05 indicates a minor significance (*) while p ≤ 0.01 corresponds to significant (**) and p ≤ 0.001 to strongly significant (***) differences. The test was followed by an all pairwise Dunn’s multiple comparison procedure as a post hoc test. The chronification of the arthritis was determined for each animal on the basis of its individual score by using the following definition: If the lowest score observed after the abatement of the acute phase (usually observed between d40 and d50) increased again by ≥ 8 points the animal was assumed to develop a chronic arthritis. For each experimental group the share of animals developing a chronic arthritis was determined and statistically significant differences in the incidence of the chronification in the groups were evaluated by again using the Kruskal-Wallis test.

### 2.4 Determination of alpha 1-acid glycoprotein

As a specific marker for arthritis alpha 1-acid glycoprotein (α1AGP) was quantified in the blood plasma of the rats during the acute arthritic phase. Briefly on d21 plasma was prepared from the blood routinely gained from the animals (see 2.5), heparinised (200 U/0.5 ml) and centrifuged for 10 min at 2000 g and room temperature. Afterwards the supernatant (plasma) was carefully removed and immediately frozen at -80°C till analysis. For the α1AGP quantification a corresponding enzyme-linked immunosorbent assay (ELISA) was used according to the manufacturer’s instructions. Absorbance was red at 450 nm and 570 nm reference wavelength with a Tecan Sunrise ELISA reader (Männedorf, Switzerland). Data analysis was accomplished using the Magellan™-Software (version 7.1; Tecan, Männedorf, Switzerland). Concentrations of α-1-AGP in the samples were calculated by reference to the appropriate standard curve. From the obtained values for each group the mean and the standard error of mean for the α1AGP concentration (ng/ml) in the plasma was determined. The results from the groups were compared by applying a one-way analysis of variance (ANOVA) and the Holm-Sidak post hoc test. Thereby p ≤ 0.05 indicates a weak significance (*) while p ≤ 0.01 corresponds to significant (**) and p ≤ 0.001 to strongly significant (***) differences.

### 2.5 Histological staining of the paws

After sacrifice of the animals on d155 the paws were taken for histopathological analysis and fixed overnight in 4% ice-cold formaldehyde. After rinsing the paws in PBS for 24 hours they were decalcified in Osteosoft® for eight weeks. After dehydration and embedding in paraffin, sections of 6 μm thickness were cut from the metacarpus of the forepaws by using a microtome (Leica SM 2010R, Nussloch, Germany) and mounted on SuperFrost Plus® slides (Menzel-Gläser, Braunschweig, Germany) for histological staining. Deparaffinized and rehydrated histological sections were stained as follows: 5 min Nuclear Fast Red (0.1% w/v), 5 min tungstophosphoric acid solution (5% w/v), 8 min Aniline blue—Orange G (0.1% w/V and 0.3% w/v, respectively). Between the single staining steps the sections were always shortly rinsed in distilled water. After the staining they were shortly differentiated in 96% ethanol, followed by dehydration in isopropyl alcohol and coverslipping with Euparal for analysis. Analysis of slices was carried out by means of a Nikon Eclipse TE2000-E microscope (Nikon Instruments Europe B.V., Düsseldorf, Germany) equipped with a Nikon Digital Sight DS-U1 camera (Nikon Instruments Europe B.V., Düsseldorf, Germany). Pictures were captured and processed with NIS-Elements BR 3.2 software (Nikon Instruments Europe B.V., Düsseldorf, Germany).

### 2.6 Blood purification and Myeloperoxidase activity determination

Every two weeks about 500 μl of blood were taken from the retrobulbar venous plexus of the animals under anaesthesia (see 2.3). The blood was collected in small sample tubes containing 200 U Heparin, stored at 4°C in the dark with gentle agitation and analysed within 24 h. For the quick depletion of the erythrocytes from the blood samples and a subsequent staining of the MPO-positive leukocytes for flow cytometry analysis a previously published protocol was used with slight modifications [37]. Briefly, for each blood sample 100 μl were transferred to a 15 ml tube and two subsequent hypotonic lysis steps were performed by adding 2 ml Millipore water and, after 60 s, 5 ml PBS (10 mM, pH 7.4). Between and after the two hypotonic lysis steps the remaining intact cells were pelleted by centrifugation for 6 min at 450 x g and 22°C. After the second centrifugation the cell pellet was dissolved in HBSS buffer supplemented with 1.26 mM calcium ions. The latter buffer was prepared on a daily basis and adjusted to pH 7.4. The purified leukocyte samples were then incubated with 10 μM APF for 30 min at 37°C. Afterwards 700 μM H_2_O_2_ were added and the samples were incubated for another 60 min at 37°C. Stock solutions of hydrogen peroxide were prepared immediately before usage in cold Millipore water and determined by using ε_240_ = 43.6 M^−1^ cm^−1^ [34]. All concentrations are final ones. Afterwards the samples were centrifuged for 6 min at 400 x g and 22°C and the obtained cell pellet was dissolved in 500 μl HBSS. The samples were immediately analysed by flow cytometry by using a FACSCalibur flow cytometer, BD Biosciences, Franklin Lakes, NY, USA supplemented with a 488 nm argon laser. Thereby the neutrophils were identified in the observed mixed blood cell fraction on the basis of their specific size and granularity and the APF-derived fluorescence within this region was detected by using channel 1 (530 ± 15 nm). In each measurement 2000 events were detected.For data analysis the software Flowing Software 2.4.1 by Perttu Terho was used. From the obtained fluorescence intensity distribution the geometric mean of the APF-derived fluorescence intensity was determined as a parameter for the chlorinating MPO activity. For each animal this value was normalised to the geometric mean obtained on d0. From these data the mean and the standard error of mean for the average peroxidase activity was determined for each experimental group. Again for the comparison of the data between the several experimental groups the Kruskal-Wallis test was applied with (*) indicating weak significant (p ≤ 0.05) differences while p ≤ 0.01 corresponds to significant (**) and p ≤ 0.001 to strong significant (***) differences.

### 2.7 Stopped-flow-spectroscopy

The direct interaction between EGCG and active MPO redox intermediates was studied by performing stopped-flow kinetic measurements. Thereby an SX-18MV apparatus from Applied Photophysics, Leatherhead, United Kingdom was used to follow the reaction of epigallocatechin (EGC) with preformed Compound I of human MPO. All measurements were performed in 100 mM phosphate buffer, pH 7.0 at 22°C. Briefly, Compound I of the enzyme was pre-formed in the aging loop by incubating native MPO with a 12.5-fold excess of freshly prepared H_2_O_2_ for 50 ms [38]. Afterwards buffer (negative control) or EGC was added and spectral changes were recorded for up to 20 s at logarithmic time intervals by using a diode array detector. Final concentrations were 2 μM MPO, 25 μM H_2_O_2_ and 5–160 μM EGC. The latter was used instead of EGCG as the gallate moiety is most likely split off during cellular uptake of the compound. For the determination of apparent bimolecular rate constants for the one-electronic oxidation of EGC by MPO Compounds I and II measurements with 1 μM MPO, 12.5 μM H_2_O_2_ and 5 -160 μM EGC (final concentrations) were performed under pseudo-first order conditions. Thereby again Compound I was pre-formed from MPO and H_2_O_2_ in the aging loop. After 50 ms the flavonoid was added and changes in the absorbance at 456 nm (Compound II) were followed by using a photomultiplier detector. Within 20 s 1000 points were recorded at logarithmic time intervals. Data from three to four independent measurements were averaged for each substrate concentration. From the initial increase in the absorbance at 456 nm and its subsequent decrease *k*_obs_ values were calculated by using double exponential function. A re-plot of these data against the EGC concentration yielded a linear dependence. The apparent second-order rate constants for the reaction of EGC with Compound I and II, respectively were obtained from the corresponding slopes. Thereby the given standard deviations correspond to the R^2^ value obtained during the linear plot of the experimental data.

## Results

### 3.1 Clinical assessment of arthritis symptoms—scoring

#### 3.1.1 Pristane-induced arthritis

**Fig 1:**
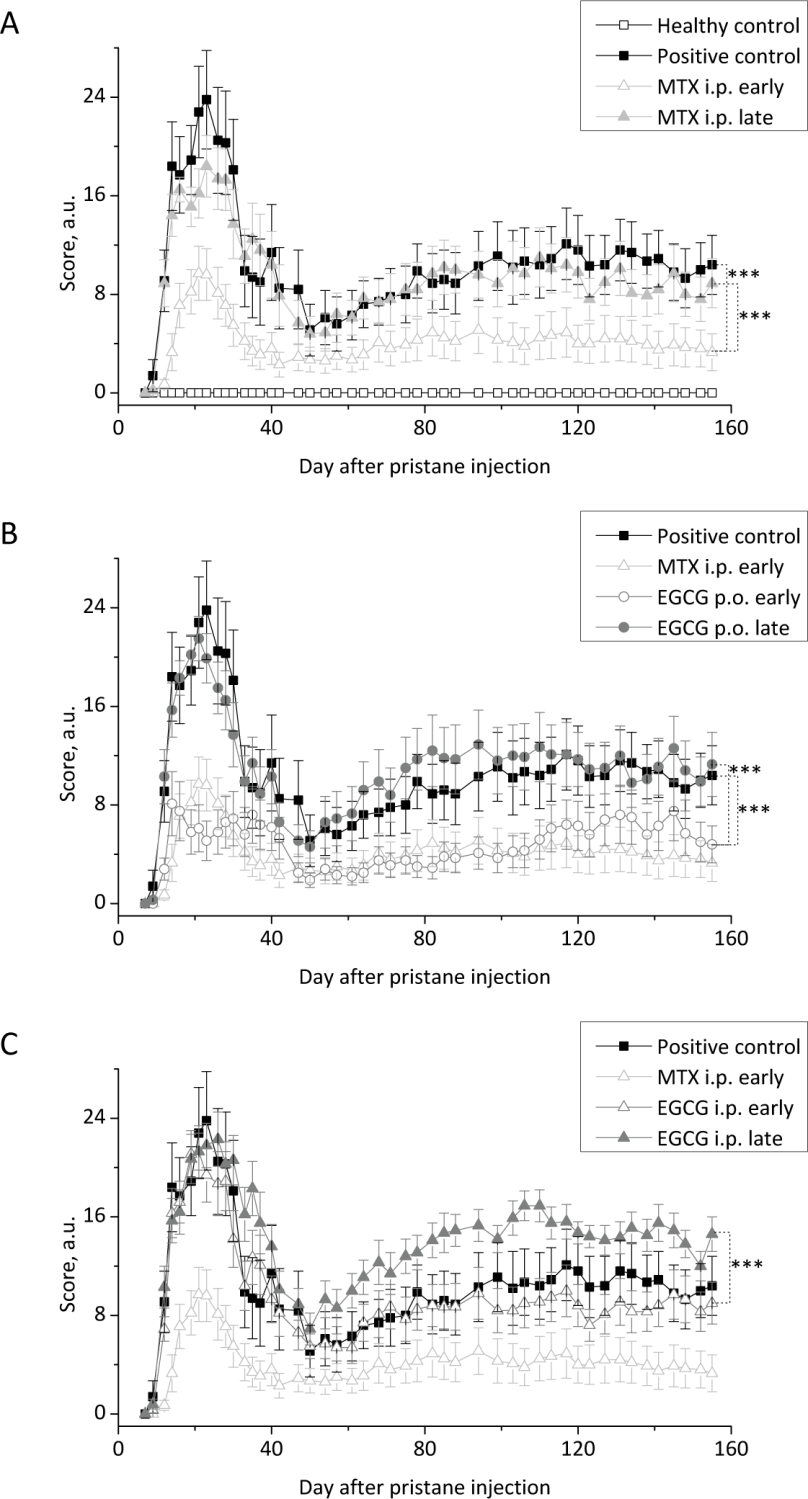
Clinical score of the paws of DA rats. While all data correspond to one experiment the experimental groups are displayed in three diagrams for reasons of comprehensibility. PIA was induced on d0 in all female DA rats except the healthy control group (empty squares; n = 5). The application of pristane led to an acute joint swelling followed by a short intermediate recovery and the subsequent development of chronic arthritic symptoms. For each treatment group the mean score of the rats for each scoring day is given with the SEM (Kruskal-Wallis test followed by Dunn’s Post Hoc test). **(A):** While the positive control (filled squares; n = 14) received saline, the treatment control received 0.05 mg MTX/kg via injection on five consecutive days. Thereby the treatment started either on d1 (light grey empty triangles; n = 15) or individually when the animals reached a score of five (light grey filled triangles; n = 14). The early treatment with MTX i.p. (treatment control) inhibited the joint swelling and redness significantly both in the acute and in the chronic phase while a late application of this treatment had no effect. **(B):** EGCG (0.1% w/v) was continuously given via drinking water again starting on d1 (grey empty circles; n = 10) or on the day the animal reached a score of five (grey filled circles; n = 10). The early treatment with EGCG p.o. considerably inhibited the joint swelling and redness in the acute and in the chronic phase just while a late application of EGCG had no effect. **(C):** 10 mg EGCG/kg were injected on five consecutive days starting either on d1 (grey empty triangle; n = 15) or on the day the animal reached a score of five (grey filled triangle; n = 15). The i.p. application of EGCG had no beneficial effect on the course of the disease and, upon late application, even led to a significantly stronger joint swelling and redness in the chronic phase.

As illustrated in Fig 1A, in the healthy control (empty squares) we never observed any joint swelling as a sign for arthritis. In contrast to that, in the positive control (filled squares, pristane injection followed by saline treatment) while no significant joint swelling was observable within the first 9 days after pristane injection (score: 1.4 ± 1.3) on d12 a significant increase in the average scoring of the group was observed (score: 9.1 ± 2.5), indicating the development of the pristane-induced arthritis (PIA). The plateau of the acute phase of the disease was observed between d14 and d30 with the highest mean score determined on d23 (23.8 ± 4.0). Afterwards the score declined until about d50 (5.1 ± 2.1), indicating the abatement of the acute phase. Yet, from about d57 (score: 5.6± 2.2) a slow but steady increase in the score was observed, reflecting the onset of the chronic arthritic phase. On d78 a score of 9.9 ± 2.2 was determined which did not change significantly until the end of the experiment on d155 (score: 10.4 ± 2.4). The highest value in the chronic phase was observed on d131 (score: 11.6 ± 2.5).Within the animals of the positive control (n = 15) and the rats who received a late treatment with either MTX i.p. (n = 10), EGCG p.o. (n = 10) or EGCG i.p. (n = 15) only one animal of the positive control and one of the MTX i.p group did not develop any arthritic symptoms after pristane injection. Thus, an incidence of at least 96.4% was achieved. By considering all 95 pristane-treated animals an incidence of even 97.9% would be obtained. The named two animals were excluded from the experiment.

#### 3.1.2 Treatment with methotrexate

As also shown in Fig 1A, five daily applications of methotrexate (MTX) from d1 after pristane injection (light grey empty triangles, early MTX treatment) significantly attenuated both the acute and the chronic phase of the PIA: In the acute phase a maximum score of 9.7 ± 2.2 was observed on d21. Afterwards the values declined more rapidly and to a bigger extent as compared to the positive control: On d42 a score of 2.3 ±1.0 was observed and till the end of the studies the values never exceeded a score of 5.1 ± 1.9 (d94). Thus, it can be concluded that the early treatment with MTX strongly inhibits the acute inflammatory phase of PIA and nearly completely abolishes the subsequent chronic phase. A global comparison of the values obtained from the positive control and the group treated with MTX on d1 yielded a strong significant difference between the score values. Thus this group was used as a treatment positive control to estimate the effects observed in the different EGCG-treated groups (Fig 1B and 1C). In striking contrast to the early MTX injections upon application of methotrexate to the animals (again 5 daily doses) after they reached a score of five (late treatment, light grey filled triangles) neither the acute nor the chronic phase of the disease could be influenced. No significant differences to the positive control but again a strongly significant difference to the early treatment with MTX was found.

#### 3.1.3 Treatment with Epigallocatechin gallate

Surprisingly, upon permanent oral application of EGCG (Fig 1B, 0.1% (w/v)) in the drinking water the observed scores strongly resemble those obtained for the MTX-treated groups: The late application (grey filled circles) did not lead to significant changes in the joint swelling and redness observed during the acute and the chronic phase of PIA as compared to the positive control. Yet if the oral administration of the flavonoid started the first day after pristane injection (early administration, grey empty circles) scores not higher than 8.1 ± 2.6 (d14) were observed during the acute inflammatory phase. On d23 (when the highest value was observed in the positive control) the score even dropped to 5.1 ± 1.6. In addition, after the values went down to 1.9 ± 0.6 (d50) after the acute phase, in the chronic phase a much lower increase in the scores was observed as compared to the positive control. The highest value was observed on d145 (score: 7.5 ± 2.4) which is well comparable with the quite low values observed in the chronic phase after early methotrexate administration. Like for MTX, a comparison of the data yielded strongly significant differences between the EGCG p.o. early group and both the positive control and the EGCG p.o. late group. In contrast to the beneficial effect of the (early) oral administration of EGCG, the injection of the flavonoid (i.p., five doses of 10 mg/kg body weight) did not show any beneficial effect on the PIA (Fig 1C): In the acute phase both the early (grey empty triangles) and the late (grey filled triangles) injection did not decrease the scoring values as compared to the saline-treated positive control. For the EGCG i.p. early group the subsequent decrease in the scores as a sign for the termination of the acute phase as well as the onset time (d50 – d60) and the severity of the chronic arthritic phase were also comparable to the saline-treated control. In contrast to that, the late i.p. administration of EGCG led to an even faster onset of the chronic phase: While on d57 the score was still relatively low in the positive control (score: 5.6 ± 2.2), a much higher arthritis score (8.6 ± 1.5) was found in the EGCG i.p. late group. Moreover, in the latter group much higher values were observed during the chronic phase (maximum score on d106/110: 16.9 ± 1.2/1.3) which exceeds the values obtained for the saline-treated control at the same time by about 60%. Thus the late injection of EGCG did not alter the acute phase of PIA but led to a stronger development of arthritic symptoms in the chronic phase.

#### 3.1.4 Chronification of the arthritis

**Fig 2:**
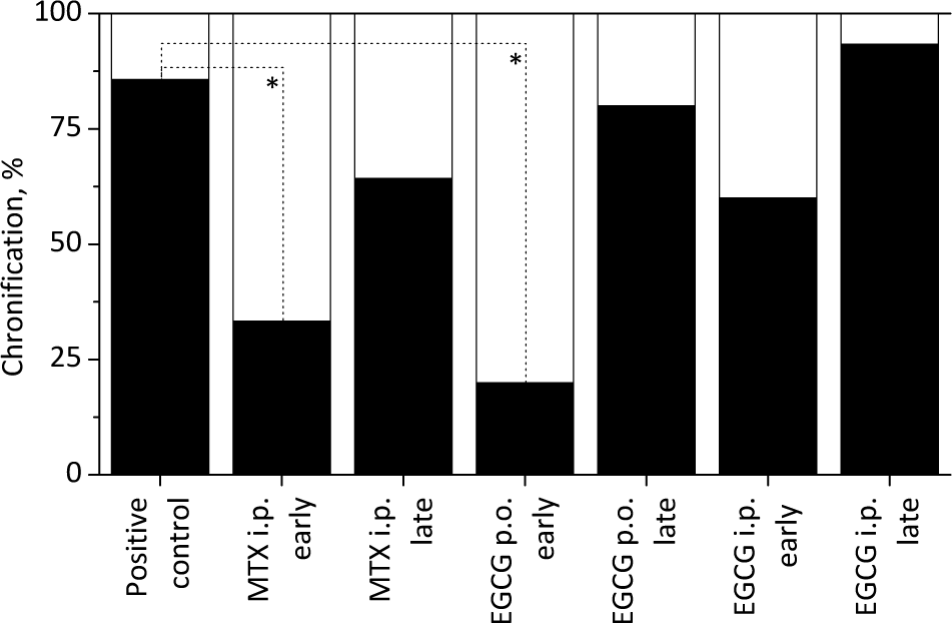
Chronification of the PIA in DA rats. The data were taken from the animal experiment displayed in Fig 1. For each animal the lowest score during the remission phase (d40–d50) was determined. If this value subsequently increased by eight or more the development of a chronic arthritic phase in the animal was assumed. The relative amount of animals which became chronic within the different pristane-treated experimental groups is shown in black. Only the early i.p. application of MTX or the early p.o. administration of EGCG led to a significantly lower amount of chronic arthritic animals. Significant differences between the experimental groups were tested by applying the Kruskal-Wallis test. Thereby (*) corresponds to p values ≤ 0.05.

For each individual animal a chronification of the pristane-induced arthritis was assumed if the lowest score observed after the abatement of the acute arthritic phase (usually between d40–d50) increased by ≥ 8 points. The suitability of this definition is proven by the fact, that despite one animal from the MTX i.p. late group all animals with acute arthritic symptoms showed a strong decrease in the scoring values between d28 and d61 (not shown). For the named animal only a moderate decrease of the score was observed during this period, which, however, strongly increased during the chronic phase. Thus the criteria defined for a chronification were also fulfilled in this case. As shown in Fig 2, for the saline-treated positive control a chronification was observed in 12 of the 14 individuals, which implies the development of chronic arthritic symptoms in nearly 86% of the animals. Yet, both in the MTX early group and in the EGCG p.o. early group a significantly lower percentage of chronification was observed (33.3% and 20.0%), proving the results stated above. In contrast, upon later application of these compounds (MTX i.p. late and EGCG p.o. late) as well as by early i.p. administration of the flavonoid no significant reduction of the amount of animals with chronic disease symptoms was observed. Upon late i.p. administration of EGCG the degree of chronification even slightly increased (93.3%) as compared to the saline control, underlining well the stronger increase of the scoring values in this group in the chronic phase.

#### 3.1.5 Onset of the arthritis

We also checked whether the early i.p. treatment with MTX or the early p.o. treatment with EGCG had an influence on the onset of PIA. Therefore the time for the first appearance of arthritic symptoms (score of one) was determined individually for each animal. From these data the mean day for the disease onset in the single experimental groups was determined. Yet, as illustrated in S1 Fig, only for the early i.p. treatment with MTX a significantly later onset of the disease (mean value: d14) as compared to the positive control (NaCl treatment, mean value: d12) was found. The value was also significantly higher as compared to the early i.p. treatment with EGCG which confirms the ineffectiveness of this treatment. In contrast, between the data for the early i.p. treatment with MTX and for the early p.o. treatment with EGCG no significant differences were found. Thus it can be guessed that in the latter treatment group at least a tendency for the later onset of PIA occurred which would be in line with the lower scoring (see 3.1.3) and chronification (see 3.1.4) values observed in this experimental group.

### 3.2 Determination of additional arthritis markers

#### 3.2.1 Determination of alpha 1-acid glycoprotein

**Fig 3:**
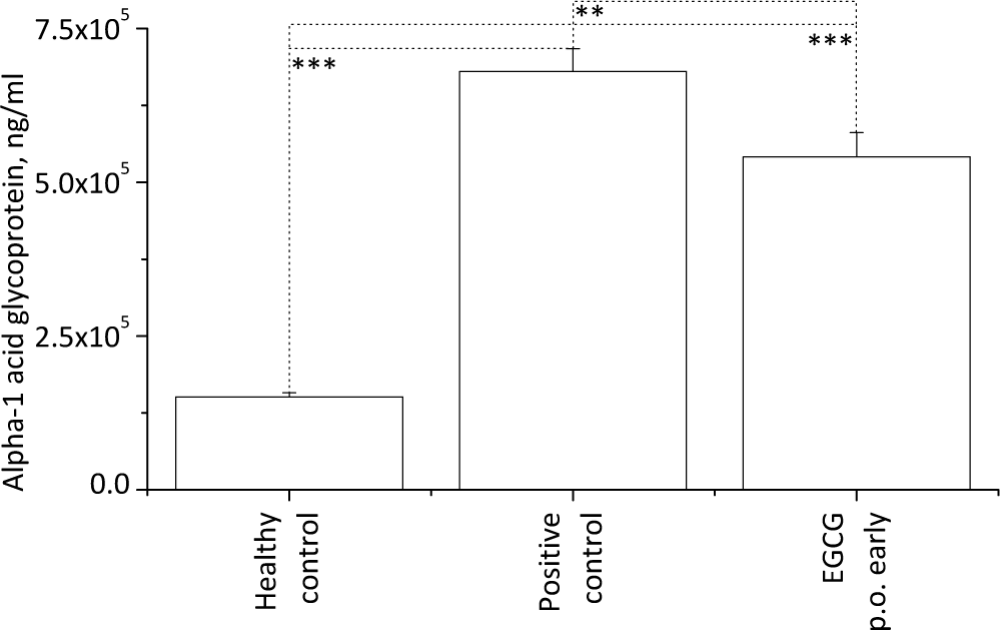
Plasma concentrations of α1AGP on d21 after pristane injection. The acute-phase protein was quantified in blood samples taken on d21 from animals of the healthy negative control (no pristane injection, n = 5), the saline-treated positive control (n = 14) and the rats treated with EGCG p.o. starting on d1 (n = 10). For experimental details see Fig 1. From the blood samples plasma was obtained and stored at -80°C till analysis via ELISA. Significant differences were determined by applying a one-way ANOVA followed by a Holm-Sidak test. Thereby (***) corresponds to p values ≤ 0.001 and (**) corresponds to p ≤ 0.01. While in the healthy control only about 150 μg/ml of the protein were found, in the positive control the amount of α1AGP was about 4.5 times higher. Yet in the EGCG-treated group the protein levels were only elevated 3.5-fold.

As an endogenous arthritis marker the amount of alpha 1-acid glycoprotein (α1AGP) was determined in the blood plasma of the animals on d21 (average maximum of the acute arthritic phase). As shown in Fig 3, in the healthy control (no pristane injection) the amount of this protein (151.1 ± 6.7 μg/ml) was significantly lower as in the groups where the oil had been injected. Yet the about 4.5 times higher value observed in the saline-treated positive control (680.0 ± 37.3 μg/ml) was again significantly reduced in the experimental group which was treated orally with EGCG starting from d1 (541.5 ± 39.2 μg/ml). These data indicate a lower inflammatory response in the latter group and nicely reflect the milder phenotype in the rats treated with EGCG p.o. from d1 as compared to the saline-treated animals.

#### 3.2.2 Histological assessment of joint destruction

**Fig 4:**
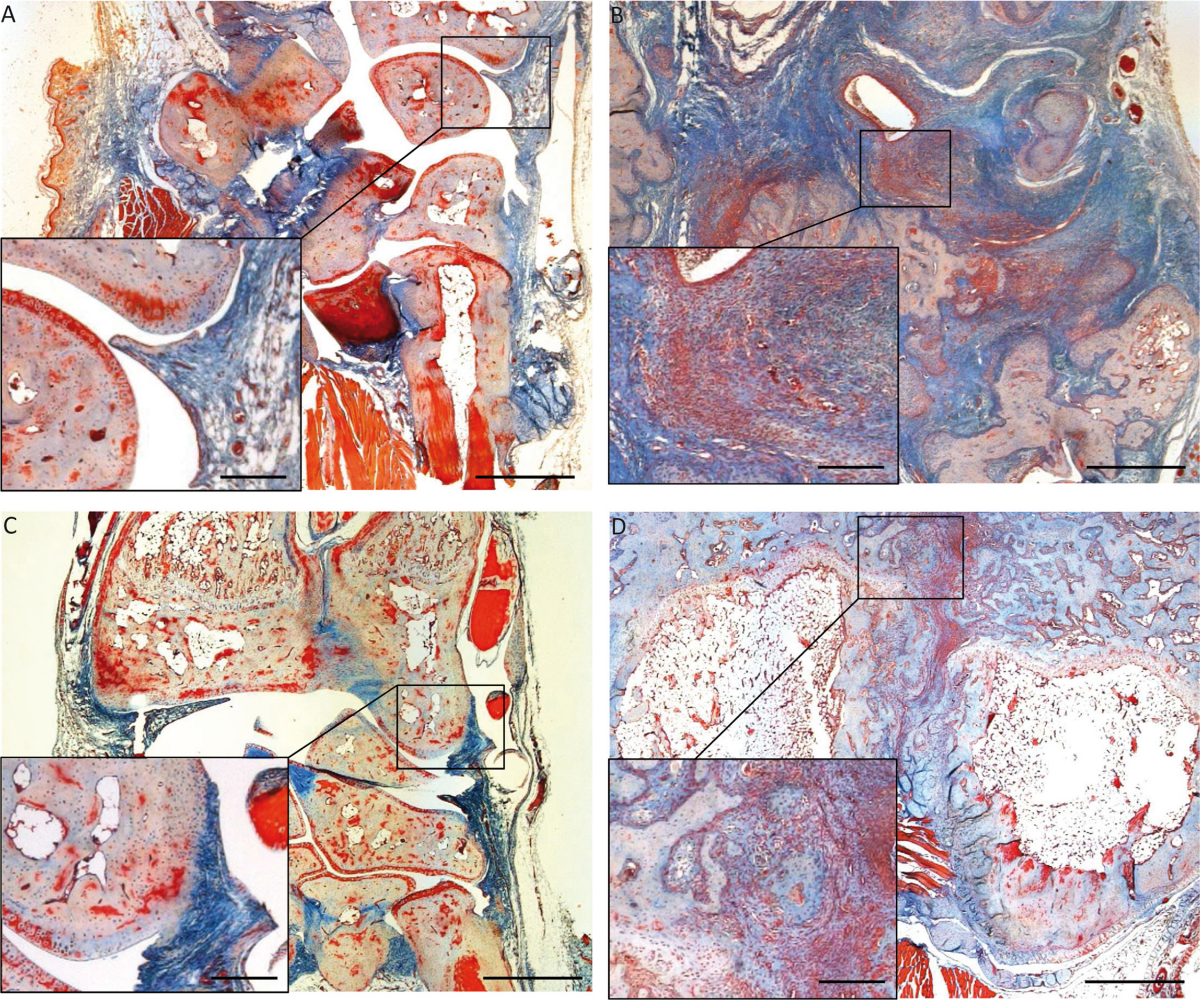
Histological analysis of metacarpal sections on d155 after pristane injection. The displayed pictures are representative examples for tissue sections obtained from healthy animals (A), from the saline-treated control group (B) and from EGCG treated groups (C: EGCG p.o. early and D: EGCG i.p. late). For experimental details regarding arthritis-induction and treatment see Fig 1. In all cases the metacarpus section of the forepaw was stained with Nuclear Fast Red, Aniline blue and Orange G. (A): In the healthy control group no signs for joint swelling and destruction were observed. Different tissue types (bone cartilage: blue, muscle: red, skin: red-orange) were clearly distinguishable. (B): In contrast, in the positive control a massive joint swelling was observable. Moreover, especially the bone and cartilage tissue was considerably destructed (dark blue regions). (C): Only mild cartilage destruction was apparent in the metacarpi of rats early and orally treated with EGCG whereas rats from the EGCG i.p. late treatment group (D) mostly showed patho-histological signs of arthritis very similar to the saline-treated positive control (B). The scale bars correspond to 1 mm (overviews) and 250 μm (inserts).

While the induction of arthritic symptoms in the pristane-treated animals was already extensively addressed by applying a clinical scoring system to quantify the swelling and redness of the paws (see. 3.1) and confirmed by quantifying α1AGP as an inflammatory blood marker at climax of clinical symptoms (see 3.2.1) at the end of the experiment, we also prepared tissue sections from the paws for histological analysis. As illustrated by the representative examples shown in Fig 4, in the healthy animals (Fig 4A, no pristane-injection) no swelling and destruction in the metacarpus was observed. Moreover, the particular tissue types (bone/cartilage/connective tissue: faint blue, muscle: red, skin: orange-red) were clearly separated from each other and showed their specific morphological structure. In contrast, the example taken from an animal of the positive control group (Fig 4B, pristane injection, saline treatment) showed a massive swelling of the metacarpus. Moreover, especially the bone and cartilage tissue was massively destructed and single bones of the metacarpus were histologically not separable (dark blue regions). Although not visible at the given magnification (scale bar of overviews: 1 mm; scale bar of inserts: 250 μm) a more detailed analysis of the latter sample also revealed a massive infiltration of leukocytes into the tissue (not shown). Fig 4C shows the metacarpus of a rat with mild signs of cartilage destruction illustrating that the early oral treatment with EGCG (pristane injection, treatment with EGCG p.o. early) attenuated the severity of arthritic symptoms, whereas the late intra-peritoneal treatment with EGCG (Fig 4D, pristane injection, treatment with EGCG i.p. late) was not able to ameliorate destruction and inflammation of PIA. In summary the histological analysis nicely reflect scoring values observed in the single experimental groups.

### 3.3 Blood MPO activity determination as a predictive marker for inflammation

#### 3.3.1 Basal chlorinating MPO activity on d0

**Fig 5:**
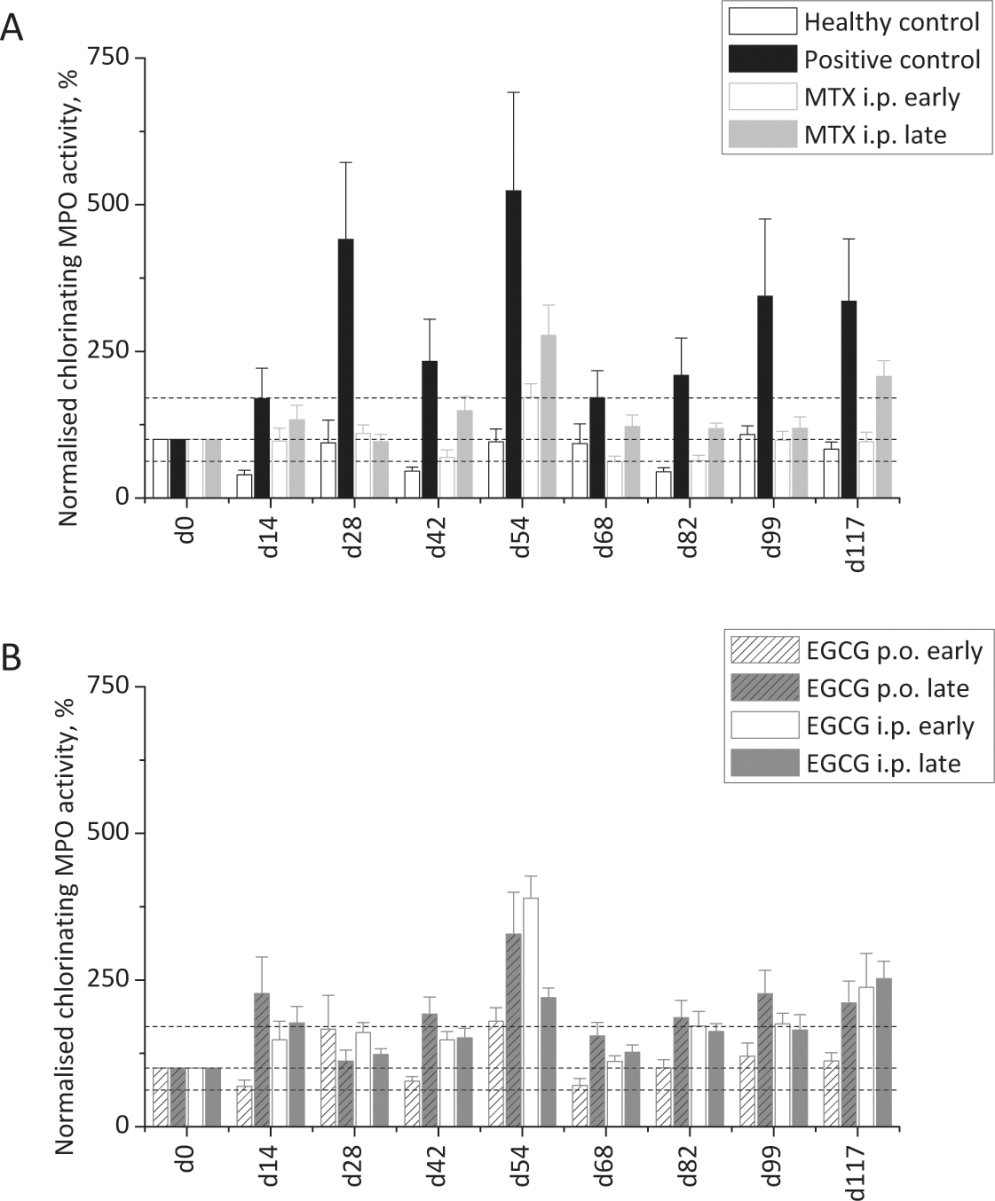
Chlorinating MPO activity in the time course of pristane-induced arthritis. For each animal the geometric mean of the APF-dependent fluorescence intensity distribution was determined as a sign for the HOCl-producing MPO activity of the blood neutrophils. **(A):** While in the healthy control group (n = 5) relatively stable values for the MPO activity were found throughout the experiment in the saline-treated positive control (n = 14), a strong increase in the HOCl production especially in the acute and in the chronic disease phase was detected. In the group with early MTX treatment (n = 15) the therapeutic effect of the drug was nicely reflected by considerably lower MPO activity values. **(B):** As the application of EGCG only reduced the arthritic symptoms upon early oral administration (n = 10), in this group also a strong reduction of the MPO activity values was observed. In the other EGCG treatment groups the HOCl production recurrently exceeded the range of normal MPO activity determined on d0 (dashed lines). Yet the differences were never statistically significant.

As a marker for the inflammatory state we repeatedly determined the myeloperoxidase activity in the neutrophils of the rats. For this analysis blood was collected from the animals under short-time anaesthesia via punctuation of the retrobulbar venous plexus. The blood samples were heparinised and neutrophils were isolated within 24 h by using a standardised protocol. By applying the HOCl-specific dye APF only the chlorinating activity of the enzyme was addressed during the subsequent flow cytometry analysis. Thereby by analysing the data obtained on d0 (before pristane injection) we observed strongly differing basal MPO activities within the several groups which, however, also emerges from the different sizes of the experimental groups. The averaged fluorescence value obtained by considering all animals (99.8 RFU) was set to 100%. By considering the relative mean value and the relative standard deviation observed in the single experimental groups this value never exceeded 170.75% (healthy control group) or fell below 62.62% (EGCG p.o. late group). These values were set as the limits for the “physiological range” of the chlorinating MPO activity while following the MPO-derived HOCl production in the blood samples during the course of the disease (dashed lines in Fig 5).

#### 3.3.2 MPO activity in the control groups

The data obtained during the repeated analysis of blood samples from the single experimental groups until d117 (Fig 5A) were individually normalised to d0. Moreover, in the graph the relative deviation of the MPO activity observed in the groups on d0 is shown (dashed lines). In the healthy control group (white columns with black outline) the chlorinating MPO activity was always quite comparable to d0 with the lowest value observed on d14 (39.5 ± 7.9%) and the highest value determined on d99 (108.5 14.6%). This illustrates well that no notable inflammatory reaction was induced in the animals in the absence of pristane and corresponds with the fact that no arthritis symptoms were observed in this experimental group throughout the whole experiment. In striking contrast to the healthy control animals in the positive control (black columns, pristane injection, saline treatment) starting on d14 considerably elevated values were found for the chlorinating MPO activity (161.5 ± 51.9%) meaning an activation of this enzyme in the neutrophils of the animals as a sign for inflammation. This reflects well the scoring data (see 3.1) observed in this group which started to significantly increase on d12. Thus the chlorinating MPO activity seems to be a good marker for the onset of inflammatory reactions. The value determined for d28 (441.1 ± 131.1) strongly exceeds the values of the healthy control group and nicely reflects the maximum of the acute arthritic phase which, according to the scoring values, took place between d14 and d30. Even the abatement of the acute phase till d50 was reflected by the MPO activity data: On d42 a value of only 232.9 ± 71.8% was determined. During the chronic phase of the disease the values determined for the HOCl-production by MPO rose again. Thereby, while the scoring of arthritic symptoms revealed an onset of the chronic arthritic phase at about d57, the MPO activity already reached a maximum on d54 (539.9 ± 167.5%) showing again that this marker reflects early events of an inflammatory reaction. Moreover while the joint swelling and redness was relatively stable during the chronic phase (see 3.1) the MPO activity data considerably fluctuated: On d68 (171.3 ± 45.8%) and on d82 (209.2 ± 63.5%) the obtained values were nearly within the borders of the normal enzyme activity in healthy animals (see dashed lines). Yet, on d99 (344.4 ± 131.3%) and on d117 (335.6 ± 106.0%) again higher values were found. Maybe these fluctuations reflect the recurring flare-up and subsiding of inflammatory reactions during the chronic phase of the PIA. In summary, regarding the positive control the determination of the chlorinating MPO activity is well in line with the scoring data: The onset, maximum and abatement of the acute arthritic phase as well as the onset of the chronic arthritis can be followed. Moreover during the latter phase the MPO data seem to indicate a wavelike course of the underlying inflammatory reaction. Yet during the statistical comparison between the healthy and the positive control (see S1 Table) we never found significant differences, most likely due to the small number of animals in the former experimental group.

#### 3.3.3 MPO activity under methotrexate treatment

The usability of the chlorinating MPO activity as a marker for the inflammatory events taking place during PIA is also illustrated by the blood analysis data obtained from the experimental groups treated with MTX (Fig 5A): If the drug was injected from d1 after disease induction (early treatment, white columns with light grey outline) the obtained MPO activity data were quite comparable to those observed in the negative control. This holds for the onset of the acute phase (d14) and its maximum (d28) as well as for its decline (d42) and the onset of the chronic arthritic phase (d54). In fact, the relative MPO activity values never exceeded 171.6 ± 23.5% (d54) and thus were always in the range of the normal activity values obtained on d0 (dashed lines). These results are in perfect line with the clinical score where animals of this group developed much weaker acute and chronic arthritic symptoms as compared to the positive control (saline treatment).The scoring data obtained after the late treatment with MTX (grey columns, drug injection after the individual score of the animals was ≥ 5) showed no significant difference to the positive control, indicating that this late treatment did not alter the development of an acute and a chronic arthritic joint swelling. Again these results are nicely reflected by the data obtained from the MPO activity analysis in the blood of the animals: On d14 (onset of the acute phase) as well as during its abatement (d42) the values obtained from the MTX i.p. late group were higher as compared to the MTX i.p. early group. Yet the values in the former group were still within the range of the MPO activity observed in healthy animals (dashed lines) and on d28 (maximum of the acute phase) were quite comparable to the values in the latter group (early MTX treatment). Thus in this case the MPO activity data misleadingly suggest a therapeutic effect of the late MTX treatment while no change in the arthritic symptoms (joint swelling and redness) was observed in this group. During the chronic phase the MPO activity data in the MTX i.p. late group are always higher than in the corresponding early treatment group. Yet they only exceed the range of physiological enzyme activity (dashed lines) on d54 (277.4 ± 51.6%) and on d117 (207.3 ± 26.8%). Still as they follow the wavelike pattern also observed in the saline-treated positive control (see 3.2.2), they suggest the development of a chronic arthritis in this experimental group. The latter is well in line with the scoring data which did not differ between the MTX i.p. late group and the saline-treated positive control group. In summary, the MPO activity data obtained from the animals treated with methotrexate nicely reflect the therapeutic effect of the drug upon early injection while the lack of this effect upon later treatment with MTX is only partially reflected by the MPO activity measurements.

#### 3.3.4 MPO activity in the EGCG treatment groups

In Fig 5B the MPO activity data obtained from the experimental groups treated with epigallocatechin gallate are summarised. Among these groups only the animals treated orally with the flavonoid (via the drinking water) starting on d1 (early application) showed significantly reduced joint swelling scores as a sign for an anti-rheumatic effect of EGCG. In line with these scoring data the determined MPO activity data were nearly always (except d28) the lowest in this experimental group (white dashed columns with grey outline). Only on d28 (maximum of the acute phase) and on d54 (onset of the chronic phase) the values slightly exceeded the range of normal MPO activity in healthy animals (dashed lines) with values of 166.1 ± 57.8% and 179.7 ± 23.1%, respectively. In contrast to the EGCG p.o. early treatment the late oral application of the flavonoid (grey dashed columns with grey outline) or the early (white columns with grey outline) or late (grey columns with grey outline) injections of the compound did not attenuate the acute and chronic arthritic symptoms, as determined by clinical assessment of arthritic symptoms (scoring of swelling and redness, see 3.1.2). These results are again reflected by the MPO activity measurements. Especially on d14 (onset of the acute phase), on d54 (onset of the chronic arthritis) and during the latter phase (d82, d99, d117) the MPO activity data obtained from these groups did not only exceed those of the EGCG p.o. early group but also the range of MPO activity in healthy animals (dashed lines). The highest values were observed on d54 with top values in the EGCG i.p. early group (389.4 ± 38.1%) which are quite in range with the values obtained from the saline-treated control at this day (523.9 ± 167.5%). Yet while in the latter group the MPO activity was also high on d28, in the three therapeutic inefficient EGCG treatment groups surprisingly low MPO activities were found. In summary, regarding the treatment of PIA in the rats with EGCG the considerably milder course of the arthritic disease after the early oral application of EGCG is nicely reflected by the MPO activity measurements. The absence of a therapeutic effect upon later oral application or injection of the flavonoid is also, at least partially, reflected by the MPO activity measurements as higher enzyme activities were found in these experimental groups. Yet it has to be stated that during the comparison of the MTX and EGCG treatment groups among each other or to the controls (see S1 Table) we sometimes observed statistically significant differences which, however, did not follow a clear pattern. This can again be attributed to the small animal numbers in some experimental groups (e.g. in the healthy control).

### 3.4 Promotion of myeloperoxidase activity by epigallocatechin

**Fig 6:**
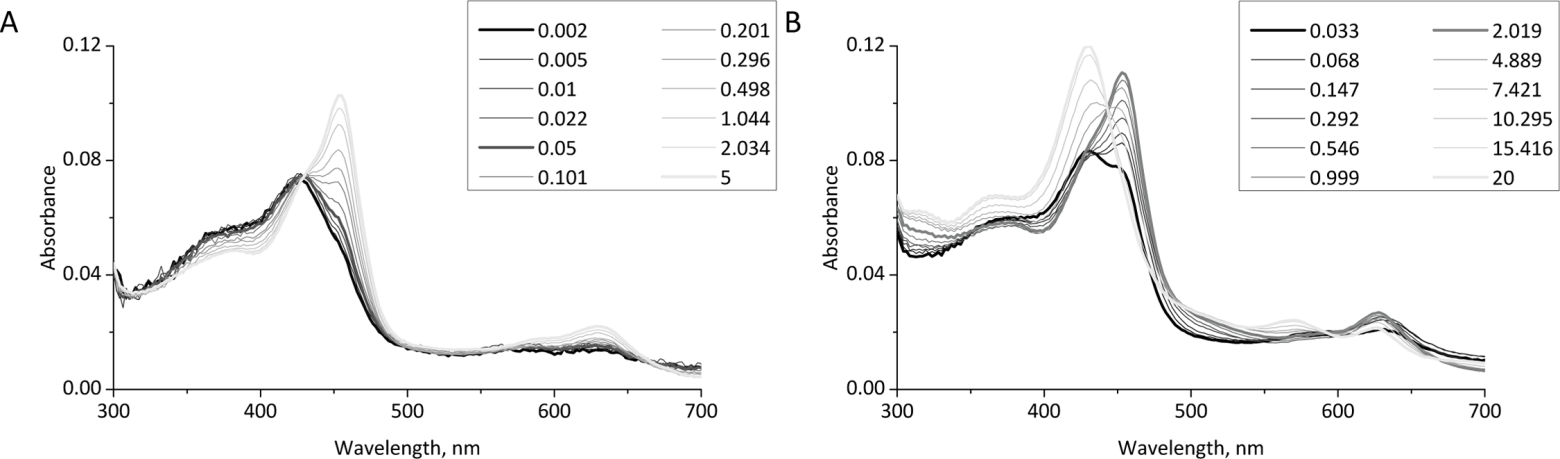
Spectral transitions of MPO Compound I by EGC. All measurements were performed at 22°C in 100 mM phosphate buffer, pH 7.0. Final concentrations of MPO and H_2_O_2_ were 2 μM and 25 μM, respectively. Representative spectra from at least three independent experiments are shown. The indicated time points were selected from 50 to 100 recorded spectra. Compound I was pre-formed by incubating the enzyme with a 12.5-fold H_2_O_2_ excess for 50 ms. **(A):** In the absence of EGC a slow H_2_O_2_-derived transition to compound II was observed within the 5 s. **(B):** However, in the presence of 20 μM EGC (final concentration) this Compound II formation was considerably faster and followed by a second transition where native MPO is regenerated from Compound II.

For (–)-epicatechin (EC) it has been shown that this flavonoid efficiently reacts with both Compound I and Compound II of MPO following the peroxidase cycle of MPO [39]. The chlorination cycle of MPO, i.e. the production of HOCl, includes ferric MPO and Compound I. However Compound I is easily converted to Compound II by one-electron donors and H_2_O_2_ and thus the latter enzymatic redox intermediate is typically the dominating redox intermediate in biological fluids, especially under inflammatory conditions [40]. Compound II cannot oxidize chloride but can be efficiently be reduced by EC to ferric MPO thereby regenerating the HOCl production by MPO [41]. Because of the structural similarity between EC and EGCG we assume that the latter flavonoid may also be easily one-electronically oxidized by MPO Compound I and II. Therefore independently from the animal experiments we also performed stopped-flow kinetic experiments studying the direct reaction of Compounds I and II of MPO with epigallocatechin (EGC), the likely intracellular form of EGCG. As shown in Fig 6A, the spectral changes observed after the addition of buffer to 2 μM MPO, pre-incubated with 25 μM H_2_O_2_ for 50 ms, show a monophasic transition from Compound I to Compound II. While the first spectrum recorded after about 2 ms (bold black) is characteristic for Compound I (Soret maximum at 428 nm) after 50 ms (bold grey) a slight shoulder formation at about 450 nm takes place, indicating the formation of Compound II. The last spectrum recorded after 5 s (bold light grey) shows a complete transition to Compound II with typical absorbance maxima at 454 nm and 628 nm and a broad shoulder at about 600 nm [42,43]. Especially the latter spectral detail allows a clear discrimination from other MPO redox states like e.g. low-spin complexes [44]. The isosbestic point at about 430 nm proves a direct reaction from Compound I to Compound II [42]. This slow H_2_O_2_-derived transition (second-order rate constant: 4.4 x 10^4^ M^-1^ s^-1^ at pH 7.0 and 15°C) is well known from the literature [42]. However, after the addition of 20 μM EGC (Fig 6B) this transition was considerably faster: The first spectrum (bold black) recorded after 33 ms already shows a considerable formation of Compound II as indicated by the formation of absorbance maxima at 454 nm and 628 nm and a shoulder at 600 nm. After about 2 s (bold grey) this first reaction is completed. Within the next 18 s, a second spectral transition takes place which shows the re-formation of native MPO. Accordingly the absorbance maxima at 454 nm and 628 nm and the shoulder around 600 nm decreased while new maxima at 428 nm and 568 nm rose, accompanied by isosbestic points at 442 nm and 587 nm [43]. The last spectrum recorded after 20 s indicates a complete regeneration of native MPO. In the absence of EGC (only MPO and H_2_O_2_) Compound II reduction was never observed, even at longer measuring times (not shown).

**Fig 7:**
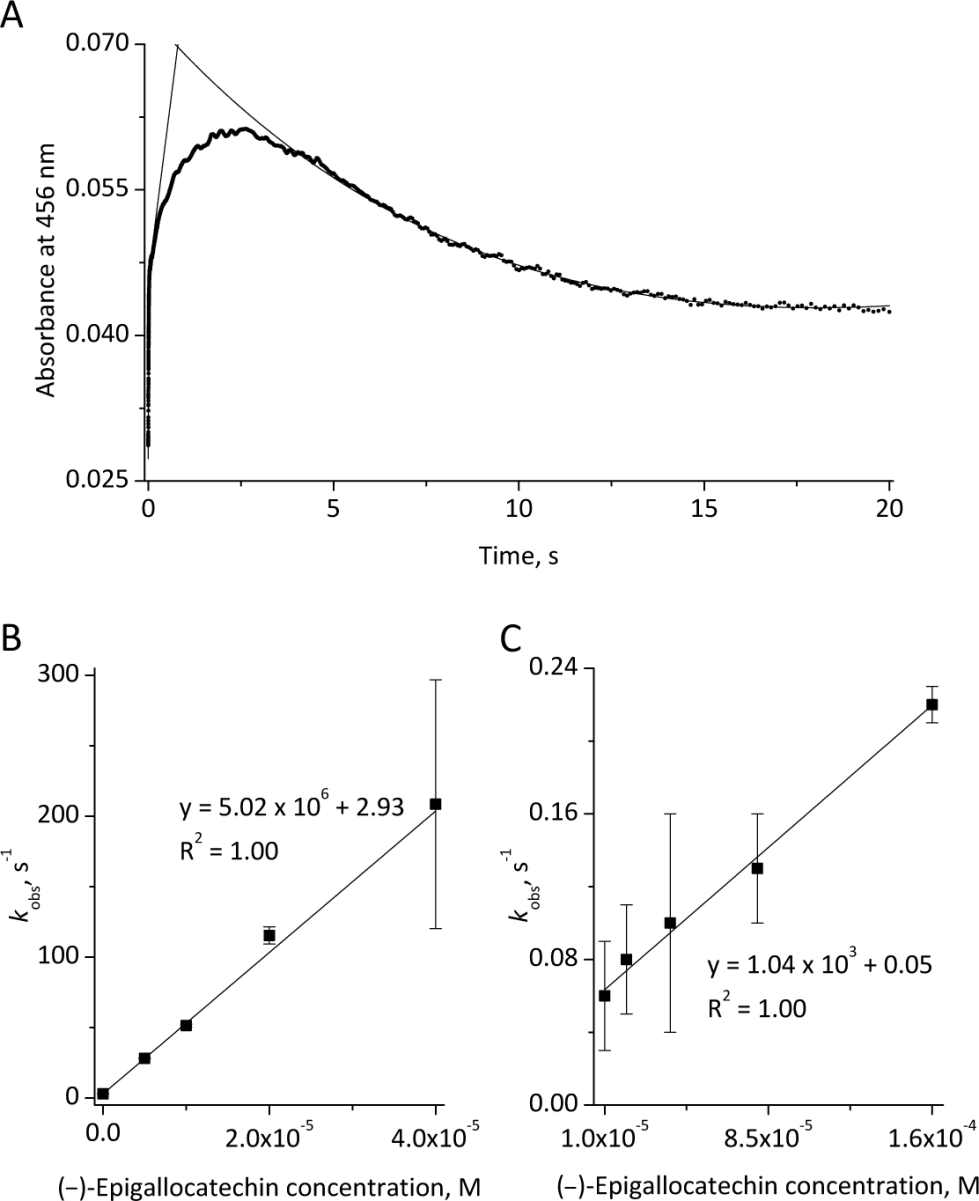
EGC-derived formation of Compound II and regeneration of native MPO. Experimental conditions are given in Fig 6. **(A):** As illustrated for 20 μM EGC time traces recorded at 456 nm are suitable to follow both transitions. By using fitting functions *k*_obs_ values were determined and re-plotted against the flavonoid concentration. **(B):** For the formation of Compound II a second-order rate constant of 5.02 x 10^6^ M^-1^ s^-1^ was determined **(C):** for the subsequent regeneration of native MPO a rate of 1.04 x 10^3^ M^-1^ s^-1^ was determined. The time traces **(A)** used for the determination of *k*_obs_ values represent averaged curves from three to four independent measurements. The linear dependence between the *k*_obs_ values and the EGC concentration is illustrated by the R^2^ values obtained during the application of linear fitting functions **(B)** and **(C)**.

In order to determine apparent second-order rate constants kinetic measurements at 456 nm and different flavonoid concentrations were performed. As illustrated in Fig 7A for 20 μM EGC typical time traces are biphasic with an initial absorbance increase at 456 nm reflecting Compound II formation followed by subsequent decrease during the regeneration of native MPO. Upon fitting the two transitions to single exponential functions *k*_obs_ values were obtained and plotted versus the flavonoid concentration (Fig 7B and 7C). From the slopes of the linear curves, apparent bimolecular rate constants were calculated to be 5.02 x 10^6^ M^-1^ s^-1^ (Compound I reduction) and 1.04 x 10^3^ M^-1^ s^-1^ (Compound II reduction), respectively (Fig 7B and 7C).

## Discussion

### 4.1 Suitability of the animal model

#### 4.1.1 Reflection of the clinical symptoms

The pristane-induced arthritis (PIA) in female *Dark Agouti* rats was used as an animal model for chronic rheumatoid arthritis (RA) in man [45]. In fact, as shown by the standardised assessment of clinical symptoms (joint swelling and redness evaluation), about two weeks after pristane injection in almost all animals the injection of pristane led to the development of an acute arthritic phase. Moreover, after a temporary decline of the symptoms, also a chronically recurring inflammation of the joints occurred. Both the high incidence of the disease as well the temporary abatement of the arthritic symptoms [46] after the acute phase and their chronic recurrence at later time points are well in line with studies of others on PIA in female *Dark Agouti* rats [7,8,45] and show that this animal model nicely reflects the recurrent joint inflammation observed at RA in man [1,2]. In line with the literature, in all animals which developed arthritic symptoms at later stages also ankylosis (joint stiffness) and joint malfunction occurred [47]. This was observable from changes in the behaviour of the animals especially within the second half of the experiment.

#### 4.1.2 Comparability of the pathological mechanisms

Also the underlying pathological mechanism, i.e. the development of a permanent pro-inflammatory systemic immune status (as reflected by increased α1AGP levels in the blood) leading to relapsing phases of joint swelling and their subsequent destruction (as evaluated by histology), is well reflected by the model. The comparability of PIA in rats to RA in man is also shown by the fact that an early application of methotrexate (MTX), a classical disease-modifying anti-rheumatic drug (DMARD) [1,2,26], delayed the onset of the disease and considerably attenuated the symptoms. In contrast, upon late MTX treatment both in the acute and in the chronic phase no clear reduction of the arthritic symptoms was observed. These results are in line with the assumption that MTX most likely acts via suppression of the innate immune response during the onset of RA [16,21].

### 4.2 Therapeutic effect of EGCG treatment

#### 4.2.1 EGCG at chronic inflammatory diseases

The flavonoid epigallocatechin gallate (EGCG) was already applied in different animal studies on chronic inflammatory diseases including RA [23,28,29]. Thereby often an attenuation of the symptoms was observed which was attributed to anti-inflammatory (e.g. inhibition of IL-6 synthesis) [23,26] and radical-scavenging properties [48] of this polyphenol. Also indirect anti-inflammatory effects like the induction of the inducible nitric oxide synthase (iNOS) were discussed as reasons for the observed therapeutic effects [29]. Yet to date EGCG was never tested for its long-term effects on RA as the used animal models, unlike PIA in rats, only lead to an acute inflammatory response in the animals [22]. Moreover, no systematic study was undertaken to evaluate the role of the time and manner of EGCG application on its anti-rheumatic effects.

#### 4.2.2 Dependence on the time and mode of EGCG application

In our study we were able to show that upon early and continuous oral application of the polyphenol therapeutic effects were achieved which are similar to those of the early injection of MTX: Both in the acute and in the chronic phase of the disease strongly reduced scoring data as well as a markedly decreased degree of chronification were observed. Even a tendency for the later onset of the arthritic symptoms was found. The orally applied EGCG concentration (0.1% w/v) is well in the range with that used in comparable studies [28,29]. These results are remarkable as MTX is still used as a gold standard both in human RA therapy and in animal arthritis models [21,27]. Moreover, as both MTX and biologicals used for RA therapy are not only sometimes ineffective but also show manifold adverse effects [25], the reported results may provide a basis for alternative therapeutic strategies. Throughout the experiment we observed a daily uptake of about 19 ml drinking water per rat which means 19 mg flavonoid in the EGCG p.o. groups. This means to a daily dose of about 100–160 mg EGCG per kg. Concentrations up to 500 mg/kg/d have no toxic effects on small rodents [26]. As revealed by studies on the oral administration of radioactively labelled EGCG to rats the flavonoid shows a peak blood concentration about 24 h after administration [49] which confirms the suitability of the daily application chosen in our study. Thereby the bioavailability of EGCG in the blood is reported to be in the range of 0.3% [49] which means about 0.3–0.48 mg flavonoid /kg in our study. In contrast to the results stated above upon late oral application no beneficial effect on the course of the disease was observed. These results were also reflected by marked differences in the degree of joint destruction observed upon histological analysis (see Fig 4**(C)** and4**(D)**) and indicate an effect of EGCG on the immunological events during disease onset, suggesting that the polyphenol mainly acts via the suppression of the innate immune response. When applied after establishment of the disease the polyphenol was unable to combat the mutual interaction between the innate and the adaptive immune system which leads to chronically recurrent arthritic joint inflammations. In fact, in several animal models for chronic inflammatory diseases the early application of EGCG was shown to especially dampen the acute response of the innate immune system [28,29], preventing, thus, a prolonged pro-inflammatory state, autoimmune reactions of the adaptive immune system and a subsequent disease chronification. In line with our results other studies with EGCG and animal models for chronic inflammatory diseases (e.g. atherosclerosis) often (but not always [26]) also only showed beneficial effects of the flavonoid after oral application but not upon injection [23,28,29]. While it was suggested that the additional stress for the animals caused by the latter treatment may play a role [28] we assume that the different pharmacokinetics upon injection may play a role: The metabolism of EGCG in the digestive tract of the rats may play a role: The flavonoid is metabolised to 5-(3’, 5’-dihydroxyphenyl)-γ-valerolactone by rat intestinal bacteria [49]. This product is further metabolized to 5-(5’-hydroxyphenyl)-γ-valerolactone 3’-*O-β*-glucurinide e.g. in the liver and enters the blood circulation [49]. Yet it remains unclear whether these degradation products contribute to the observed therapeutic effects of EGCG upon oral application. The polyphenol concentration in the injection solution (10 mg/kg) was comparable to the experiments of others [23].

#### 4.2.3 Effect of the early oral EGCG application on α1AGP

The assumption that EGCG, upon early oral application, mainly acts via inhibition of the acute immune response induced by pristane injection is also in line with the determination of α1AGP blood levels determined at d21 of the experiment. As already reported by others both in RA patients and in corresponding animal models the hepatic mRNA expression levels as well as plasma levels of this acute phase protein are known to be markedly elevated [50–52], making this acute-phase protein a suitable marker for the course of the disease. Accordingly, in our study the injection of pristane led to about 4.5-fold elevated α1AGP blood levels (680.0 μg/ml) as compared to the healthy control (151.1 μl), which is nicely comparable to already published data [53,54]. Thus a permanent pro-inflammatory immune status can be assumed to be induced by pristane injection.Yet upon early oral application of EGCG the blood concentration of this acute-phase glycoprotein was about 20% lower as compared to the positive control (pristane injection, NaCl treatment). While the exact physiological function of α1AGP is still unknown [50,51], the observation that antibodies against this glycoprotein react with granulocytes and monocytes suggests some immune-regulatory effects of α1AGP on the innate immune system [50]. Thus the decrease of the α1AGP blood level observed upon early and continuous oral application of EGCG provides another hint that it mainly acts via inhibition of the acute response of the innate immune system. Although we also obtained plasma samples from the other experimental groups, only for the three indicated groups we were able to properly determine α1AGP levels. So we can only guess that the level of this acute phase protein was also significantly lower in the MTX i.p. early group.

### 4.3 MPO as a novel inflammatory marker

#### 4.3.1 Repeated assessment of the chlorinating enzyme activity

The obtained results suggest, in line with recent studies, that the innate immune system and neutrophils play an important role especially during the onset of RA [11]. In order to address the systemic activation status of these cells we determined the chlorinating activity (i.e. the formation of hypochlorous acid, HOCl) of one of the most abundant neutrophil enzymes, namely myeloperoxidase (MPO). By using a standardised method the erythrocyte amount in the samples was diminished and the chlorinating MPO activity was measured by using the dye aminophenyl fluorescein (APF) [37,55]. To our knowledge such a repeated evaluation of the MPO activity whereby especially its HOCl-producing activity is addressed was not performed in an animal model before.

#### 4.3.2 Chlorinating MPO activity as an inflammatory marker

As illustrated by the analysis of blood samples from d0, the basal MPO activity showed strong individual differences. Still it was possible to define a range for the range of its physiological activity. In fact, by repeated analysis of blood samples from the healthy control group (no pristane injection) this range was never exceeded throughout the experiment. In striking contrast to this in the positive control (pristane injection, NaCl treatment) the MPO activity values clearly followed the course of the disease including onset and abatement of the acute phase and beginning of the chronic phase. Within the latter stadium the MPO activity levels seemed to follow a periodic pattern.Thus the HOCl-producing MPO activity nicely reflects the arthritic symptoms observed during the course of the experiment. In fact, expression and plasma levels of the proteins are elevated both in RA patients and in animal models for the disease [12,20,56]. It is assumed that these increased protein levels correlate with a higher HOCl-producing enzyme activity, while often only the general enzymatic activity [20] or the formation of HOCl-derived protein modifications (e.g. chlorotyrosine) [56] is measured. Moreover as illustrated by the fluctuating values observed during the chronic phase the assessment of the chlorinating activity may provide additional information about the actual systemic inflammatory status, which are not directly visible from the symptom assessment. In man RA is known to be characterized by recurring flare-ups of the immune system intermitted by phases of low immune activity [1,2,4]. The suitability of the chlorinating MPO activity as a marker for the systemic inflammatory status during RA is also reflected by the data obtained from the MTX early treatment group: In line with the observed therapeutic effect the HOCl production rates were always within the physiological range. Surprisingly, while the late MTX injection did not significantly influence the occurrence of arthritic symptoms also in this experimental group the corresponding MPO activity data were significantly lower as compared to the positive control. Maybe after establishment of the disease a reduction of the systemic inflammatory status by the drug (as reflected by the MPO activity measurements) is not sufficient to compete with the mutual interaction between the innate and the acquired immune system. Thus again the detection of the chlorinating MPO activity provides more insights into the inflammatory status than the clinical assessment of the symptoms and, thus, may represent a new biomarker to follow the course of chronic inflammatory disease [4].

#### 4.3.3 Effect of EGCG on the chlorinating MPO activity

In line with the therapeutic effect observed upon early and continuous oral application of EGCG in this group the determination of the chlorinating MPO activity almost always yielded values which were in the range of the physiological enzyme activity determined on d0. Only on d28 (acute phase) and on d54 (onset of the chronic phase) the values slightly exceeded this physiological range. All other EGCG treatment groups (late oral EGCG treatment and early/late EGCG injection) yielded higher MPO activity values which, especially during the acute phase (d14 and d28) as well as at the onset of the chronic phase (d54), considerably exceeded the range of physiological enzyme activity determined on d0. Still even in these experimental groups where EGCG showed no therapeutic effect the HOCl production rates were lower as compared to the positive control (pristane injection, NaCl treatment) suggesting a certain anti-inflammatory effect of the flavonoid. Still it has to be clearly stated that while the analysis of the chlorinating MPO activity turned out as a good marker to distinguish between healthy animals and untreated animals with PIA (saline-treated positive control) as well as to detect efficient anti-rheumatic treatments (MTX i.p. early and EGCG p.o. early), the HOCl production by MPO turned out to only partially reflect the clinical scoring data observed in less effective treatment groups.

### 4.4 Link between MPO and the therapeutic effect of EGCG

#### 4.4.1 Role of MPO at rheumatoid arthritis

Given the huge role of the innate immune system not only during the onset of RA but also during the chronification of the disease [11,19] there is also growing evidence for a role of MPO and its enzymatic products not only as a marker but as an active player during the pathogenesis of this disease [9]: The protein is secreted during degranulation and/or NETosis of stimulated neutrophils [17] and, thus, may contribute to the formation of autoantibodies during the chronification of RA [9]. A role of MPO-derived ROS in cartilage and bone destruction during later stages of RA was shown by using MPO knockout (MPO^-/-^) mice as in the absence of the protein less tissue damage occurred [20]. Yet during these studies no difference in the cytokine profile and an even elevated CD4^+^ T cell response were observed in the MPO^-/-^ animals, indicating that the enzyme is also involved in the suppression of adaptive immune responses [20].Thus, MPO contributes to local tissue damage during RA but may also have a protective systemic role during disease chronification [20]. In fact, as has been recently shown, the chlorinating activity of the enzyme may contribute to the inhibition of pro-inflammatory neutrophil and macrophage signalling and, thus, limit long-lasting systemic pro-inflammatory conditions [40,57,58]. The observation that MPO is inactive in the synovial fluid of RA patients provides a further hint for the role of its enzymatic activity to counteract chronic pro-inflammatory conditions [56]. Recent findings suggest a negative correlation between oxidative burst capacity and arthritic symptoms: In DA rats the polymorphism of *neutrophil cytosolic factor 1* (*Ncf1*), a gene essential for NADPH oxidase complex formation and ROS production in neutrophils, increased the susceptibility to PIA [59]. In fact, upon induction of the ROS production a lower T cell activation was observed in the *Ncf1*^DA^ rats [59].

#### 4.4.2 Chlorinating MPO activity during inflammation

An interesting aspect regarding the immunological role of the HOCl production by MPO comes from the fact that several well-known anti-inflammatory compounds (e.g. the plant-derived polyphenol (–)-epicatechin [41,60]) as well as drugs like ascorbic acid [61] and acetaminophen [62] are well known for their ability to regenerate to chlorinating MPO activity which is often impaired at inflammation: Several conditions at inflammatory loci including excess hydrogen peroxide, high nitric oxide levels [40,63] are known to inhibit the HOCl production by driving MPO to Compound II, an enzymatic redox intermediate which is not able to catalyse the two-electron oxidation of chloride [64]. The above stated substrates regenerate the HOCl production by reduction of Compound II to the ferric MPO state [40]. We thus hypothesize that the well-known anti-inflammatory effects of (–)-epicatechin [41,60] and the named drugs may, at least in part, be linked to their activity-promoting effect on the HOCl production by MPO [40].

#### 4.4.3 *In vitro* reactivity of EGCG with MPO Compound I and II

The effect of one-electron donors on the chlorinating MPO activity depends on two main factors: (I) The currently prevailing redox state of the enzyme which strongly depends on the actual (patho-)physiological conditions and (II) the reaction rates of potential substrates with Compound I (*k*_2_) and Compound II (*k*_3_) of the enzyme. The ratio *k*_2_/*k*_3_ helps to estimate whether a given one-electron donor may act as an inhibitor or a promoter of the chlorinating MPO activity. For tryptophan, a ratio of 65.000 was determined [65] meaning that this amino acid suppresses the chlorinating MPO activity and, in case of accumulated Compound II, does not lead to its recovery. In striking contrast, the *k*_2_/*k*_3_ ratio of (–)-epicatechin is 4.2 [39] leading to the regeneration of the chlorinating MPO activity both at the isolated enzyme and in human neutrophils [41]. Regarding EGC in this study we determined for the first time its reactivity with MPO Compounds I and II and calculated a *k*_2_/*k*_3_ ratio of about 4800. While this ratio is not as low as observed for (–)-epicatechin it can still be guessed that under conditions of Compound II accumulation the polyphenol contributes to the regeneration of the chlorinating MPO activity. Most interestingly, salicylates (e.g. acetylsalicylic acid), one of the earliest known NSAIDs against RA, are also well-known substrates for MPO which bring back Compound II to the native enzymatic form [6,62]. Yet it has to be stated that other NSAIDs used in RA treatment are also known to promote Compound II accumulation of MPO [66,67], showing the ambivalent immunological role of MPO and its HOCl-producing activity in RA. While the studies on isolated MPO were performed with EGC the obtained results are still transferable to the application of EGCG in the rat experiments as the latter is metabolised to EGC by intestinal bacteria [49]

### 4.5 Physiological relevance

#### 4.5.1 Limitations of the animal model and the MPO activity analysis

As for any animal model the differences in the organisation of the immune system between rodents and humans also limit the comparability between PIA in rats and RA in humans [68]. For instance, blood levels of the acute phase protein α1-AGP used as a systemic inflammatory marker in this animal study are about ten times lower in humans [51]. The phenotypes and the immunological activity of neutrophils are also different between man and rodents which was shown to play an important role by assessing the suitability of animal models for RA [9]. Still the observation that the higher intake of pristane by fishermen results in a higher prevalence for RA suggests a link between the pathology of PIA in rats and RA in man [69].Furthermore it has to be stated that the described method is not suitable to determine changes in the amount of neutrophils in the blood as the applied erythrocyte depletion method for neutrophil enrichment obscures e.g. elevated neutrophil levels which are likely to occur during inflammation [11]. In fact, a relative amount of 39.9 ± 19.9% neutrophils was observed during the flow cytometry analysis, independent of the sampling time and the experimental group. Moreover also e.g. a possible activation of the cells during RA [17] is not perfectly reflected by the MPO activity data as the gate defined during the flow cytometry analysis includes resting as well as activated neutrophils. Here the application of additional markers would provide more insights and may help to figure out why the chlorinating MPO activity investigated in this study did not always reflect the inflammatory status of the animals as determined e.g. by α1AGP plasma levels or the scoring of clinical symptoms. In fact, in previous studies where we applied the same leukocyte enrichment method we observed that neutrophils from mice and rats, unlike human cells, do not show considerably increased FSC- or SSC-values during flow cytometry analysis [37].

#### 4.5.2 Transferability of the results obtained on EGCG and MPO

In our animal study the early and continuous oral application of EGCG had a strong therapeutic effect. Yet these results are not directly transferable to humans as the applied polyphenol concentration (0.1% w/v) would mean a daily intake of about 500 mg EGCG in man which is at least five times more than the normal dose [26,29]. Yet such high concentrations can be reached by intake of the flavonoid as a food supplemental as e.g. illustrated by clinical studies on the pharmacological effect of EGCG [70]. However, there are also reports about adverse effects resulting from the continuous intake of such high concentrations of this green tea polyphenol [71]. In this study on PIA in rats the repeated determination of the chlorinating MPO activity turned out as a suitable marker to follow the underlying systemic inflammatory reaction. Therapeutic effects of MTX and EGCG were also reflected by the MPO activity values. Since neutrophils are much less abundant in mice and rats than in man [68,72] we expect that the determination of the HOCl-producing MPO activity could provide a suitable new marker for clinical studies on RA in man. This fact, the small amount of blood necessary for this analysis [37] and the assumption that the innate immune system, neutrophils and MPO may play a bigger role in RA as classically assumed [6,9,56] provide further arguments for considering the activity of this enzyme in future studies on rheumatoid arthritis.

#### 4.5.3 Role of neutrophils at chronic inflammatory diseases

As already stated neutrophils do not only participate in the onset and solidification of RA [6,9] as well as in the tissue destruction observed at later stages of the disease [10,11] but there are also indications for a physiological role of these cells in limiting systemic inflammatory reactions occurring at RA [20,40]. As proven by new studies thereby the secretion of serine proteases by the cells most likely play a role which lead to the degradation of pro-inflammatory cytokines and chemokines [73,74]. Thereby the formation of nuclear extracellular traps (NETs) appears to be essential as a failed NETosis leads to chronic inflammatory conditions [73]. This protease-dependent anti-inflammatory role of neutrophils was shown to be independent of the oxidative burst capacity of the cells [74]. Still as the NET formation is known to be ROS-dependent [73] these results may suggest another explanation for the anti-inflammatory role of MPO activity at chronic inflammatory diseases like RA.

## Supporting Information

S1 Fig Onset of the PIA in DA rats. PIA was induced on d0 in all female DA rats except the healthy control group. The data were taken from the animal experiment displayed in Fig 1. The onset of the disease was assumed at a score of one and individually determined for each animal. From these data the average day of disease onset was calculated. Thereby the displayed box plots show the median (bold line), the 25% and 75% quartile (box boundaries) as well as the highest and lowest values (circles) determined within the several experimental groups. In the positive control (saline treatment) as well as in both early EGCG groups (p.o. and i.p.) the mean value for the PIA onset was d12 while in the MTX i.p. early group a disease onset on d14 was determined. Significant differences between the experimental groups were tested by applying the Kruskal-Wallis test. Thereby (*) corresponds to p values ≤ 0.05.
(TIF)

S1 Table Dunn‘s Multiple Comparison Test for the comparison of the mean MPO activity values determined for the experimental groups at the indicated days. (DOCX)

## Abbreviations

α1-AGP: α 1-acid glycoprotein
ANOVA: analysis of variance
APF: aminophenyl fluorescein
CFA: complete Freund's adjuvant
CIA: collagen-induced arthritis
DA: Dark Agouti
DMARD: disease-modifying anti-inflammatory drug
DMSO: dimethyl sulfoxide
EC: epicatechin
EGC: epigallocatechin
EGCG: epigallocatechin gallate
ELISA: enzyme-linked immunosorbent assay
GTP: green tea polyphenol
IgG: immune globulin G
IL: interleukine
iNOS: inducible nitric oxide synthase
i.p.: intra-peritoneal
MIP-1: macrophage inflammatory protein 1
MTX: methotrexate
MPO: myeloperoxidase
Ncf1: nuclear cytosolic factor 1
NETs: nuclear extracellular traps
NSAID: non-steroidal anti-inflammatory drug
PBS: phosphate buffered saline
PIA: pristane-induced arthritis
PMNs: polymorphonuclear leukocytes
p.o.: per os (orally
RA: rheumatoid arthritis
RFU: relative fluorescence units
SIRS: systemic inflammatory response syndrome
SLE: systemic lupus erythematosus
TNFα: tumor necrosis factor α
