# Supplementary figures and images for: Long-Term Effects of (–)-Epigallocatechin Gallate (EGCG) on Pristane-Induced Arthritis (PIA) in Female Dark Agouti Rats
Source: PLoS One. 2016 Mar 29;11(3):e0152518. doi: 10.1371/journal.pone.0152518 (PMC4811407; doi:10.1371/journal.pone.0152518)

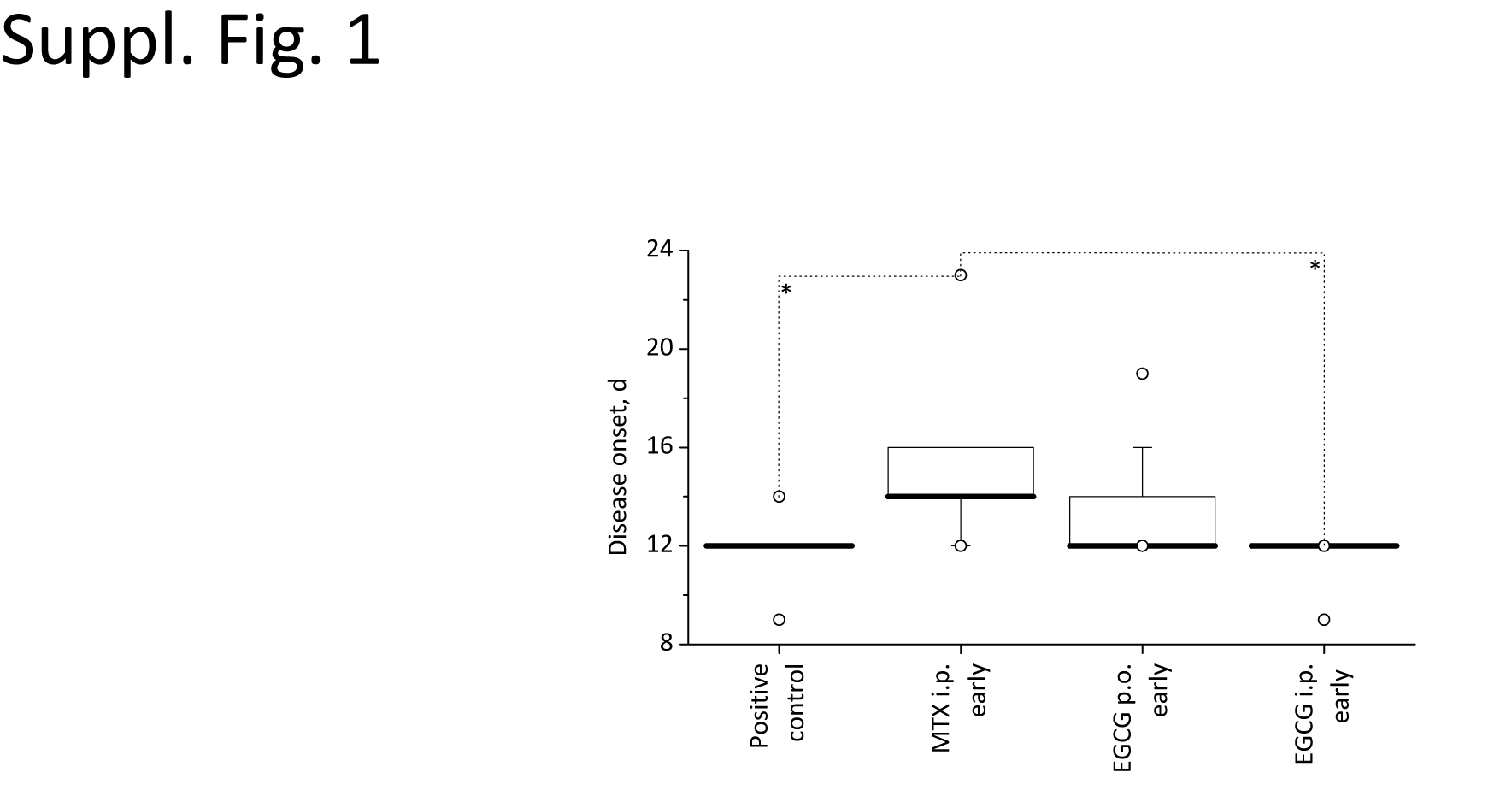

Supplement: S1 Fig — PIA was induced on d0 in all female DA rats except the healthy control group. The data were taken from the animal experiment displayed in Fig 1. The onset of the disease was assumed at a score of one and individually determined for each animal. From these data the average day of disease onset was calculated. Thereby the displayed box plots show the median (bold line), the 25% and 75% quartile (box boundaries) as well as the highest and lowest values (circles) determined within the several experimental groups. In the positive control (saline treatment) as well as in both early EGCG groups (p.o. and i.p.) the mean value for the PIA onset was d12 while in the MTX i.p. early group a disease onset on d14 was determined. Significant differences between the experimental groups were tested by applying the Kruskal-Wallis test. Thereby (*) corresponds to p values ≤ 0.05. (TIF) [file pone.0152518.s001.tif]
