# Supplementary material for: Long-Term Effects of (–)-Epigallocatechin Gallate (EGCG) on Pristane-Induced Arthritis (PIA) in Female Dark Agouti Rats
Source: PLoS One. 2016 Mar 29;11(3):e0152518. doi: 10.1371/journal.pone.0152518 (PMC4811407; doi:10.1371/journal.pone.0152518)
Supplement: S1 Table — (DOCX) [file pone.0152518.s002.docx]

Suppl. Tab. 1: Dunn‘s Multiple Comparison Test for the comparison of the mean MPO activity values determined for the experimental groups at the indicated days.

| **Compared groups** | **d14** | **d28** | **d42** | **d54** | **d68** | **d82** | **d99** | **d117** |
| --- | --- | --- | --- | --- | --- | --- | --- | --- |
| Healthy control vs. Positive control | ns | ns | ns | ns | ns | ns | ns | ns |
| Healthy control vs MTX i.p. early | ns | ns | ns | ns | ns | ns | ns | ns |
| Healthy control vs MTX i.p. late | ns | ns | * | ns | ns | ns | ns | ns |
| Healthy control vs EGCG p.o. | ns | ns | ns | ns | ns | ns | ns | ns |
| Healthy control vs EGCG p.o. late | ** | ns | *** | ns | ns | * | ns | ns |
| Healthy control vs EGCG i.p. early | ns | ns | ** | ** | ns | ** | ns | ns |
| Healthy control vs EGCG i.p. late | ** | ns | ** | ns | ns | ** | ns | * |
| Positive control vs MTX i.p. early | ns | ns | ns | ns | ns | ns | ns | ns |
| Positive control vs MTX i.p. late | ns | * | ns | ns | ns | ns | ns | ns |
| Positive control vs EGCG p.o. early | ns | ns | ns | ns | ns | ns | ns | ns |
| Positive control vs EGCG p.o. late | ns | ns | ns | ns | ns | ns | ns | ns |
| Positive control vs EGCG i.p. early | ns | ns | ns | ns | ns | ns | ns | ns |
| Positive control vs EGCG i.p. late | ns | ns | ns | ns | ns | ns | ns | ns |
| MTX i.p. early vs MTX i.p. late | ns | ns | * | ns | ns | ns | ns | * |
| MTX i.p. early vs EGCG p.o. early | ns | ns | ns | ns | ns | ns | ns | ns |
| MTX i.p. early vs EGCG p.o. late | ns | ns | *** | ns | ** | ** | ns | ns |
| MTX i.p. early vs EGCG i.p. early | ns | ns | ** | ** | ns | ** | ns | ns |
| MTX i.p. early vs EGCG i.p. late | ns | ns | ** | ns | ** | *** | ns | ** |
| MTX i.p. late vs EGCG p.o. early | ns | ns | ns | ns | ns | ns | ns | ns |
| MTX i.p. late vs EGCG p.o. late | ns | ns | ns | ns | ns | ns | ns | ns |
| MTX i.p. late vs EGCG i.p. early | ns | * | ns | ns | ns | ns | ns | ns |
| MTX i.p. late vs EGCG i.p. late | ns | ns | ns | ns | ns | ns | ns | ns |
| EGCG p.o. early vs EGCG p.o. late | ns | ns | * | ns | ns | ns | ns | ns |
| EGCG p.o. early vs EGCG i.p. early | ns | ns | ns | ns | ns | ns | ns | ns |
| EGCG p.o. early vs EGCG i.p. late | ns | ns | ns | ns | ns | ns | ns | ns |
| EGCG p.o. late vs EGCG i.p. early | ns | ns | ns | ns | ns | ns | ns | ns |
| EGCG p.o. late vs EGCG i.p. late | ns | ns | ns | ns | ns | ns | ns | ns |
| EGCG i.p. early vs EGCG i.p. late | ns | ns | ns | ns | ns | ns | ns | ns |

*: p ≤ 0.05; **: p ≤ 0.01; ***: p ≤ 0.001; ns: not significant
